# Epigenetic Programing of B-Cell Lymphoma by BCL6 and Its Genetic Deregulation

**DOI:** 10.3389/fcell.2019.00272

**Published:** 2019-11-07

**Authors:** Haopeng Yang, Michael R. Green

**Affiliations:** ^1^Department of Lymphoma and Myeloma, Division of Cancer Medicine, The University of Texas MD Anderson Cancer Center, Houston, TX, United States; ^2^Department of Genomic Medicine, The University of Texas MD Anderson Cancer Center, Houston, TX, United States

**Keywords:** BCL6, chromatin modifying genes, histone acetylation, histone methylation, B-cell lymphoma, germinal center

## Abstract

B cell lymphoma is a clinically heterogeneous and pathologically diverse group of diseases with a strong epigenetic component. The B cell lymphoma 6 (*BCL6*) gene encodes a transcription factor that is critical for normal germinal center reaction B cell development by maintaining an epigenetic and transcriptional state that is permissive for cellular proliferation and DNA damage. The activity of BCL6 can be deregulated by a variety of mechanisms and contributes to the development of B-cell lymphoma. Here we review the direct and indirect mechanisms BCL6 dysregulation in B cell lymphoma, including transcriptional and post-translational regulation of BCL6 expression and activity, and the perturbation of BCL6-regulated epigenetic programs by cooperating chromatin modifying gene mutations. We underscore the critical importance of BCL6 and its associated epigenetic programs in the development of B-cell lymphoma, and discuss avenues for the therapeutic targeting of BCL6 in this context.

## Introduction

Epigenetic alterations control gene transcription through DNA methylation and the post-translational modification of chromatin, which leads to the activation or silencing of genes. Epigenetic dysfunction has been shown to have important roles in the etiology of a range of hematological malignancies, including B cell lymphoma (Shaknovich and Melnick, 2011; Jiang et al., 2016). Most B-cell non-Hodgkin lymphomas (NHL), including diffuse large B-cell lymphoma (DLBCL) and follicular lymphoma (FL), arise from germinal center B (GCB)-cells; a stage at which B cells undergo rounds of proliferation and edit their immunoglobulins by somatic hypermutation (SHM) and class-switch recombination (CSR), followed by affinity selection via interactions with other immune cells such as follicular T-helper cells and follicular dendritic cells. The entry to, and exit from, the germinal center (GC) reaction requires large changes in gene expression that are controlled by transcription factors which recruit co-activator or co-repressor complexes that drive dynamic epigenetic and transcriptional changes. The deregulation of these molecular programs perturb the normal process of B cell development and contribute to the etiology of B cell lymphoma (Kuppers, 2005).

The B cell lymphoma 6 (*BCL6*) gene encodes a transcription factor belonging to the Broad-Complex, Tramtrack and Bric-a-brac/Pox virus and Zinc Finger (BTB/POZ) family (Ye et al., 1993) that is critical for the initiation and maintenance of GC reactions (Basso and Dalla-Favera, 2010; Hatzi and Melnick, 2014). Murine models with conditional deletion of BCL6 in B-cells fail to form GCs or produce high-affinity antibodies (Dent et al., 1997; Ye et al., 1997). The BCL6 protein has three conserved domains that are important for its function; the N-terminal BTB/POZ domain that recruits corepressors such as BCOR, NCOR1, and NCOR2, a central RD2 region that interacts with CTBP, NuRD, MTA2, and HDAC2, and a C-terminal zinc finger domain that interacts with specific DNA sequence (Chang et al., 1996; Ahmad et al., 2003; Ghetu et al., 2008; Huang et al., 2014). These interactions enable BCL6 to repress transcription via the recruitment of co-repressor complexes, which allow for the suppression of more than 1000 target genes (Ci et al., 2009; Basso et al., 2010; Basso and Dalla-Favera, 2012; Hatzi et al., 2013). These interactions are required for BCL6 activity, as point mutations in the BTB domain that prevent the corepressors binding lead to the reduced proliferation and survival of GCB cells (Huang et al., 2013). This is, in part, due to the role of BCL6 in repressing cell cycle checkpoint and DNA damage repair associated genes such as *CDKN1A* and *TP53* (Phan and Dalla-Favera, 2004; Phan et al., 2005), which allows for clonal expansion and persistence following SHM-induced DNA damage, respectively. In normal GCB cells, BCL6 also suppresses the expression of the anti-apoptotic oncogene, BCL2, via binding to its promoter region and interacting with transcriptional activator Miz1. This primes normal GCB cells for apoptosis, which is important for clonal deletion. But in DLBCL and FL, BCL6-mediated suppression of BCL2 is often lost due to the translocation of BCL2 and deregulation of Miz1 (Saito et al., 2009). The BCL6 gene is therefore the master regulator of GCB cell development and functions via the recruitment of co-repressor complexes that catalyze broad epigenetic changes.

The BCL6 gene has long been recognized as an important oncogene in B-cell lymphoma due to its direct deregulation by promiscuous translocations that place it under control of the immunoglobulin heavy-chain locus or a variety of alternative regulatory elements (Ye et al., 1993; Chen et al., 1998). However, recent studies have revealed additional mechanisms by which the expression or activity of BCL6 are deregulated by genetic alterations. Here, we will review the role of genetic alterations in altering BCL6 function in B-cell lymphoma.

## Direct Regulation of Bcl6

### Deregulation of *BCL6* Gene Expression

**FIGURE 1:**
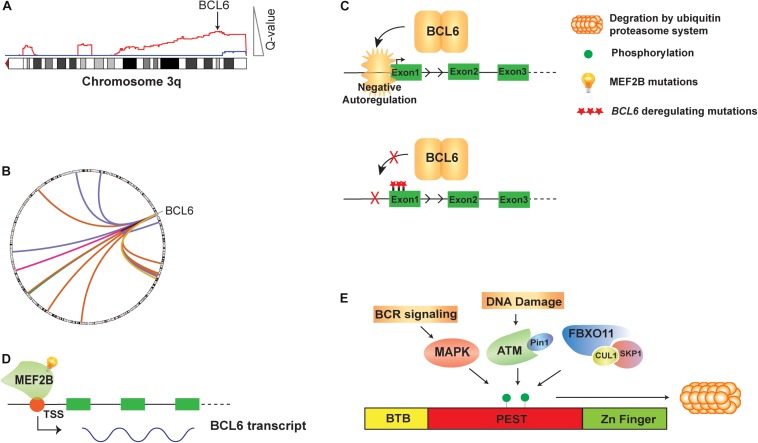
Genetic alteration and direct regulation of BCL6 in B cell lymphoma. **(A)** A schematic of 3q DNA copy number gain (red) with GISTIC Q value corresponding to DNA copy number gain is shown. *BCL6* copy gain is highlighted with arrow. **(B)** A circos plot shows translocations of *BCL6* to a variety or partner genes. **(C)** BCL6 homodimer binds to its own promoter and negatively auto-regulate its expression. Mutations on the first non-coding exon of *BCL6* disrupt this negative autoregulatory circuit by preventing BCL6 from binding its regulatory region. **(D)** MEF2B directly activates the transcription of BCL6 in normal GCB cells and mutations of *MEF2B* lead to deregulated expression of BCL6 in B cell lymphoma. **(E)** The BCL6 protein is regulated at the post-translational level by phosphorylation. Activated B cell receptor (BCR) signaling, DNA damage and SKP1–CUL1–Fbox protein (SCF) complex that contains the orphan F-box protein FBXO11 can all drive phosphorylation of BCL6 and its degradation by ubiquitin proteasome system.

BCL6 was originally identified in DLBCL as the target of frequent chromosomal translocations occurring on chromosome 3q27 (Baron et al., 1993; Kerckaert et al., 1993; Ye et al., 1993; Figures 1A,B). Subsequent studies also showed that its expression could be deregulated by off-target SHM of its promoter region (Pasqualucci et al., 2003). Unlike other genes that are aberrantly mutated such as *PIM1* or *MYC*, *BCL6* mutations are found in about 30% of human GCB cells and memory B cells, due to the physiological somatic hypermutation (Pasqualucci et al., 1998, 2001; Shen et al., 1998). However, an analysis of BCL6 mutant alleles from DLBCL tumors and normal GCB cells show a toward for mutations within the first non-coding exon of *BCL6* in DLBCL, which could disrupt its circuit of negative autoregulation by preventing BCL6 from binding its own promoter (Wang et al., 2002; Pasqualucci et al., 2003; Figure 1C). The expression of BCL6 is also regulated by the MEF2B and IRF8 transcription factors. The *MEF2B* gene is mutated in ∼11% of DLBCLs and ∼12% of FL, resulting in enhanced transcriptional activity, increased BCL6 expression, and increased proliferation of DLBCL cell lines (Figure 1D; Ying et al., 2013). As the most common *MEF2B* mutant, MEF2B^*D*83*V*^ drives lymphomagenesis and in mouse model, Mef2b^*D*83*V*^ led to GC enlargement and lymphoma development (Brescia et al., 2018). Furthermore, 85% of *MEF2B* missense mutations were located in the N-terminal conserved MADS box and MEF2 functional domains, suggesting that they may regulate BCL6 at transcriptional level. Knocking down of *MEF2B* in DLBCL cell lines led to downregulation of BCL6 expression and suppression of cell proliferation. Using a luciferase reporter assay, multiple *MEF2B* N-terminal missense mutants (D83V, Y69H, and L54P) displayed an increased transcriptional activity on the promoter region of BCL6 (Ying et al., 2013). The *IRF8* gene is also mutated at a lower frequency in FL (∼6%; Li et al., 2014). Although these mutations have not been interrogated functionally, they have the potential to also affect BCL6 expression because IRF8 binds to BCL6 promoter and initiates BCL6 expression upon GC entry (Lee et al., 2006).

### Post-translational Control of BCL6

The BCL6 protein is regulated at the post-translational level by phosphorylation (Figure 1E), acetylation and methylation. Activation of B cell receptor (BCR) signaling leads to MAP kinase-mediated phosphorylation of BCL6 protein, and subsequent degradation by the ubiquitin-proteasome system (Niu et al., 1998). DNA damage was also reported to induce BCL6 degradation by the proteasome, through ATM-dependent BCL6 phosphorylation and interaction with the isomerase Pin1 (Phan et al., 2007). The *FBXO11* gene encodes an F-box protein that functions as a component of the SKP1-CUL1-Fbox protein (SCF) ubiquitin ligase complex that targets BCL6 for degradation in a phosphorylation-dependent manner. Expression of FBXO11 results in a marked reduction of BCL6 levels, which can be mitigated by either the addition of proteasome inhibitors or introducing a dominant-negative CUL1 mutant that leads to accumulation of SCF substrates. In contrast, the depletion of FBXO11 by shRNAs induces an increase in the steady-state levels and stability of BCL6. Inactivating mutations of *FBXO11* occur in 4-9% of DLBCL tumors and impair its ability to induce BCL6 degradation, thereby contributing to lymphomagenesis through BCL6 stabilization (Duan et al., 2012). Additionally, conditional *Fbxo11* knockout mouse model displayed an increased number of GC B cells and higher levels of BCL6 protein in GC B cells (Schneider et al., 2016).

Acetylation of BCL6 by CREBBP and EP300 disrupts its ability to recruit histone deacetylases (HDACs). Co-immunoprecipitation assay showed that BCL6 interacts with HDAC2, and that this could be disrupted by either acetylation of BCL6 or by glutamine substitutions of the acetylated residues (Bereshchenko et al., 2002). Moreover, addition of HDACs inhibitor trichostatin A and/or SIRT inhibitor niacinamide displayed an increasing level of acetylated BCL6 in both normal and transformed B cells, suggesting that acetylation of BCL6 is controlled by both HDAC-dependent and SIR2-dependent pathways (Bereshchenko et al., 2002). The CREBBP and EP300 genes are recurrently mutated in FL and DLBCL, which may result in the reduced acetylation of BCL6 and associated increases in BCL6 activity (Pasqualucci et al., 2011a). Protein arginine methyltransferase 5 (PRMT5)-mediated methylation of BCL6 at R305 is also necessary for the full transcriptional repressive effects of BCL6 (Lu et al., 2018). The methylation of BCL6 is not known to be perturbed by genetic mutations, but is potentially targetable as a result of the recent development of selective PRMT5 inhibitors (Wang et al., 2018). In line with this, the PRMT5-specific inhibitor GSK591 has been shown to induce the derepression of BCL6 target genes and reduce the proliferation of DLBCL cell lines (Lu et al., 2018).

## Indirect Avenues for Unbalanced Bcl6 Activity

### Cooperation Between BCL6 and Polycomb Complexes

**FIGURE 2:**
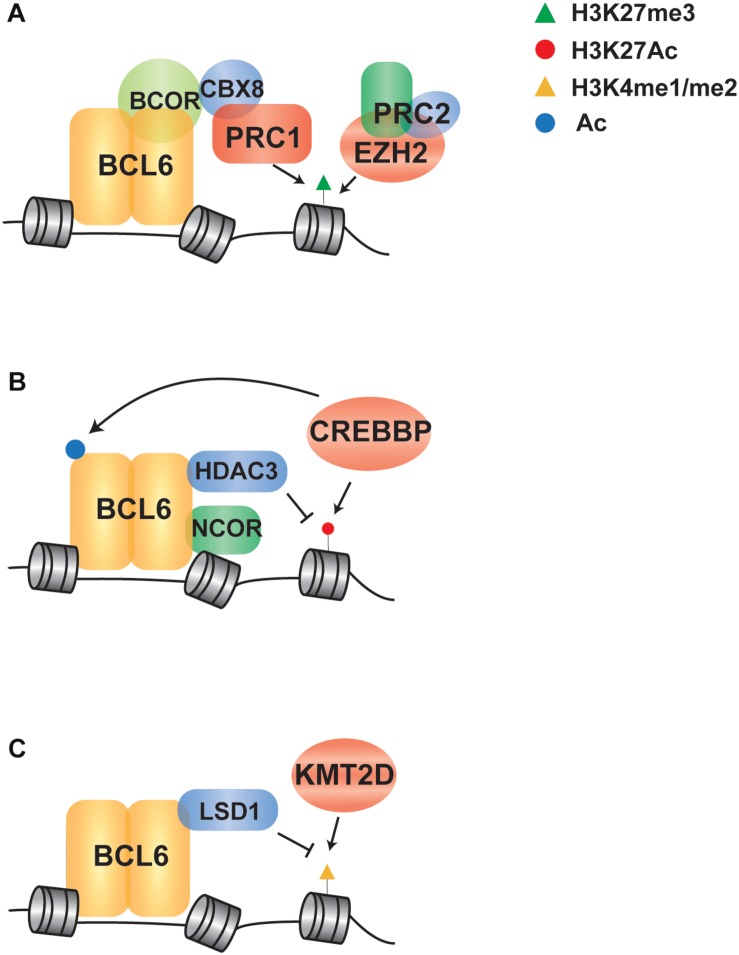
Indirect regulation of BCL6 via crosstalk with chromatin modifying genes in B cell lymphoma. **(A)** A schematic shows that EZH2 and BCL6 cooperate to recruit a PRC1/BCOR complex to repress gene expression via enhanced H3K27me3. **(B)** BCL6 represses transcription through NCOR/HDAC3 complex-mediated deacetylation of H3K27, which can be potentiated through loss of function mutations in *CREBBP* that leave HDAC3 activity unopposed. **(C)** BCL6 directly recruits LSD1 to its target genes and demethylates H3K4 to modulate enhancer functions in GC-derived lymphoma cells, in line with the role of *KMT2D* mutations in reducing H3K4 methylation in B cell lymphoma.

BCL6 cooperates with EZH2 by tethering non-canonical PRC1-BCOR-CBX8 complex that catalyze H3K27me3 and coordinate the GC phenotype through formation of bivalent chromatin domains at critical GCB cell promoters (Beguelin et al., 2016; Figure 2A). The *EZH2* gene encodes a histone H3 lysine 27 (H3K27) methyltransferase that is recurrently mutated in FL and DLBCL. Deletion or pharmacologic inhibition of *Ezh2* in murine B cells resulted in the reduction of GC formation (Beguelin et al., 2013; Caganova et al., 2013), demonstrating that it is a critical regulator of GCB cell development. Mutations of *EZH2* are always heterozygous and typically target the tyrosine 641 or alanine 677 residues within the catalytic SET domain (Morin et al., 2010; McCabe et al., 2012a). This results in a hypomorphic protein with increased activity in adding the third methyl group to dimethylated H3K27. In cooperation with the wild-type protein, which has relatively higher mono- and di-methylation activity, the presence of a hypomorphic protein results in the accumulation of trimethylated H3K27 (H3K27me3) (McCabe et al., 2012a). The expression of EZH2^*Y*641*F*^ mutant led to GC hyperplasia and promoted lymphomagenesis in cooperation with BCL2 overexpression. Mechanistically, EZH2^*Y*641*N*^ is associated with an increased H3K27me3 at gene promoter, resulting in aberrant repression of sets of genes such as *PRDM1* and *CDKN1A* (Beguelin et al., 2013). Importantly, these genes are also regulated by BCL6 and the deletion of *Cdkn1a* was sufficient to rescue GC formation in EZH2 conditional knock-out mice (Beguelin et al., 2017). These data suggest that BCL6 and EZH2 have cooperative roles in GCB cell development, and hypomorphic *EZH2* mutations may therefore contribute to the epigenetic deregulation of BCL6 target genes. Importantly, specific inhibitors of EZH2 have been developed and are currently being investigated in early phase clinical trials (Knutson et al., 2012; McCabe et al., 2012b). Clinical responses of B-cell lymphomas to one of these inhibitors, Tazemetostat (Italiano et al., 2018), are not limited to cases with hypomorphic mutations of *EZH2.* This therefore suggests that the wild-type activity of EZH2 in GCB cells, and hence the activity of BCL6, may be an additional determinant of response to EZH2 inhibition.

### Antagonism of BCL6 by CREBBP/EP300

In addition to the cooperation with polycomb complexes, BCL6 can repress transcription through complexes with SMRT (NCOR2) and HDAC3, which results in H3K27 de-acetylation (Ahmad et al., 2003; Hatzi et al., 2013; Figure 2B). Upon receiving terminal differentiation signals via CD40, which induce the expression of IRF4 and subsequently inactivate BCL6 expression, these genes can be reactivated by histone acetyltransferases that drive GC exit and B-cell terminal differentiation (Saito et al., 2007; Cardenas et al., 2017). As the second most frequently mutated chromatin modifying gene in B-cell lymphoma, the *CREBBP* gene is recurrently mutated in 65% of FL patients and 16% of DLBCL (Pasqualucci et al., 2011a; Green, 2018). CREBBP has a catalytic domain that functions as an acetyl-lysine “writer” and a bromodomain that functions as an acetyl-lysine “reader.” It acts as a transcriptional co-activator by catalyzing the acetylation of H3K18 and H3K27, as well as the acetylation of other non-histone proteins (Bedford and Brindle, 2012). The majority of *CREBBP* mutations are missense changes within the catalytic lysine acetyltransferase domain that result in a decreased in activity (Mullighan et al., 2011; Pasqualucci et al., 2011a). A minority of mutations are truncating events that eliminate CREBBP protein expression (Pasqualucci et al., 2011a), and these are more frequent in DLBCL compared to FL (Garcia-Ramirez et al., 2017). In human follicular lymphoma, *CREBBP* mutations are associated with a significant reduction of MHC class II expression on the lymphoma B-cells (Green et al., 2015). In transgenic mouse models, conditional deletion of *Crebbp* also resulted in reduced MHC class II(Hashwah et al., 2017; Jiang et al., 2017; Zhang et al., 2017), and loss of MHC class II expression was alone sufficient increase the penetrance of lymphoma (Hashwah et al., 2017). Mechanistically, BCL6 and CREBBP co-occupy many of the same enhancer elements in GCB cells and the inactivation of BCL6-mediated repression is accompanied by CREBBP-mediated gene reactivation to drive GC exit and B-cell terminal differentiation (Jiang et al., 2017; Zhang et al., 2017). Regions with reduced H3K27Ac associated with *Crebbp* deletion are enriched for BCL6 target genes, suggesting that *CREBBP* mutations may in part contribute to lymphoma by eliminating opposition to BCL6-mediated silencing of gene expression (Jiang et al., 2017). However, a role for BCL6 in MHC class II gene expression has not been previously observed, so the role of BCL6 and CREBBP opposition in this critical axis remains to be defined.

The *EP300* (aka p300) gene encodes a lysine acetyltransferase that is homologous to *CREBBP* and is also recurrently mutated in a lower frequency in B cell lymphomas (Pasqualucci et al., 2011a, b). Given the significant structural and functional similarities between *CREBBP* and *EP300* (Xu et al., 2006), they are together responsible for the global acetylation of H3K18/H3K27, and other locus-specific histone acetylation events (Xue et al., 2018). Loss of function mutations of *EP300* were found to increase proliferative potential in hematopoietic progenitor cells and the defect in hematopoiesis in EP300-null cells can be rescued by introducing an extra copy of CREBBP under control of EP300 locus (Kimbrel et al., 2009). Furthermore, conditional biallelic knock-out *Crebbp* in combination with monoallelic knock-out of *Ep300*, or vice versa, in murine B-cells showed that these acetyltransferases control unique as well as shared transcriptional targets in GCB cells (Meyer et al., 2019). The redundant targets of CREBBP and EP300 may be essential for tumor cell survival because DLBCL cell lines with truncating mutations of *CREBBP* are sensitive to knock-out or pharmacologic inhibition of EP300 (Meyer et al., 2019). Moreover, though EP300 is able to acetylate BCL6 and repress its recruitment of HDACs (Bereshchenko et al., 2002), EP300 itself is a direct target gene of BCL6 (Cerchietti et al., 2010b) and in primary DLBCL samples, BCL6 and EP300 levels are inversely correlated (Shaknovich and Melnick, 2011). EP300 and CREBBP therefore have a subset of non-overlapping functions in B-cell development and therefore likely also in lymphomagenesis.

### Crosstalk Between BCL6 and KMT2D

The histone demethylase LSD1, which specifically catalyzes demethylation of H3K4me1/2, was recently found to be required for GC formation and BCL6-driven lymphomagenesis. BCL6 directly binds with LSD1 and mediates the recruitment to its direct target genes at intergenic and intronic enhancers (Hatzi et al., 2019), suggesting that BCL6 may play a role in the directing the demethylation of enhancers marked with H3K4me1/2 (Figure 2C). Consistent with an important role for H3K4 methylation in B-cell lymphomagenesis, the *KMT2D* gene (aka MLL2) is the most frequently mutated chromatin modifying gene in FL and DLBCL(Green, 2018). This gene encodes a methyltransferase protein that catalyzes the methylation of H3K4 at promoters and enhancers, resulting in transcriptional activation; generally, promoters are marked with tri-methylation of H3K4 (H3K4me3) while enhancers are marked with mono-methylation and di-methylation of H3K4 (H3K4me1 and H3K4me2) (Calo and Wysocka, 2013). Mutations of *KMT2D* are predominantly non-sense or frameshift events that truncate the C-terminal catalytic SET domain, or less frequently missense mutations that diminish its methyltransferase activity, resulting in reduced H3K4 methylation (Zhang et al., 2015). Conditional deletion of *Kmt2d* early during B cell development in murine model has shown increased GCB-cell frequency following immunization and promotes B cell proliferation, in part due to the transcriptional changes of cell cycle and apoptosis associated genes (Zhang et al., 2015). In line with defective maturation, murine B-cells deficient in *Kmt2d* also showed a reduced frequency of class switch recombination (Ortega-Molina et al., 2015). Notably, KMT2D exerts a broad effect by controlling the expression of multiple key regulators of CD40, Toll-like, and B cell receptor signaling pathways that are also regulated by BCL6 (Li et al., 2005; Basso et al., 2010; Ortega-Molina et al., 2015). Together, these studies suggest that the mutations of *KMT2D* may also be contribute to lymphoma in part via the epigenetic deregulation of BCL6 target genes.

## Targeting Bcl6

In addition to the previously mentioned avenues for modifying BCL6 activity through PRMT5 and EZH2 inhibition, multiple direct inhibitors of BCL6 have been developed. These include BCL6 peptidomimetics, small molecule inhibitors and degraders. In 2004, a BTB binding domain peptide (BBD peptide) was designed based on the crystallographic data of BCL6 BTB–SMRT complex (Ahmad et al., 2003; Polo et al., 2004). The peptide was found to occupy the lateral groove of BCL6 and further block the recruitment of corepressors, resulting in the attenuation of BCL6-mediated transcriptional repression and the establishment of silenced chromatin state. It also abrogated BCL6 biological function in B cells and recapitulated the failure of GC formation in BCL6 null mice (Polo et al., 2004). Upon treatment of a panel of BCL6-positive and BCL6-negative cells with BBD peptide, only the BCL6 expressing cell lines showed sensitivity in growth inhibition and apoptosis to the peptide (Polo et al., 2004). Subsequently, to enhance the efficacy and protease resistance, BBD peptide was modified by shortening the sequence, adding a fusogenic motif and mutating a proline to a glycine (Cerchietti et al., 2009). Through screening on a series of lymphoma cell lines, the new retroinverso BCL6 peptide inhibitor (RI-BPI) showed the ability to selectively kill BCR-type rather than OxPhos-type cells, and to reactivate important BCL6 target genes such as ATR and TP53. Moreover, RI-BPI also displayed good inhibition in the treatment of BCR type cell line xenografted mice model and primary human BCL6 expressing DLBCL (Cerchietti et al., 2009). By computer aided drug design, low molecular weight compounds that potentially bind in the BCL6 lateral groove were also identified. Compound 79-6, which was screened from over 1,000,000 commercially available compounds, showed high binding affinity to the pocket in the lateral groove of the BCL6 BTB domain, with the Kd of 138 ± 31 mM. Additionally, this compound could induce expression of BCL6 target genes, kill BCL6-positive DLBCL cells and potently suppress tumor growth in human DLBCL xenografts models (Cerchietti et al., 2010a). Using an *in silico* drug design functional-group mapping approach called SILCS, 79-6 was modified to yield a compound with much higher affinity to the lateral groove of the BCL6 BTB domain called FX1. FX1 disrupted the formation of the BCL6 repression complex, reactivated BCL6 target genes and suppressed ABC-DLBCL cells both *in vitro* and *in vivo*, as well as primary human ABC-DLBCL specimens (Cardenas et al., 2016). Recently, a new approach for directly targeting BCL6 was reported based on a structure-based drug design (Kerres et al., 2017). Multiple proteolysis targeting chimeras with high affinity to the BCL6 BTB domain were derived to also induce ubiquitylation and proteasome-dependent degradation of BCL6. Strikingly, BCL6-degrading compounds could reactivate BCL6-repressed genes more potently than non-degrading inhibitors. They also displayed anti-proliferative effects on several DLBCL cell lines, with some IC50 values below 10 nM. However, due to the poor bioavailability, the animal study of BCL6 degrader has not been reported. Together, these findings all provide the basis for directly targeting BCL6 as therapeutic approach for B cell lymphoma, but these approaches are yet to be tested in the clinic.

## Future Directions

We are only beginning to scratch the surface of the complex epigenetic basis for B-cell lymphoma, and the role that BCL6 plays in this setting. Here we have provided an overview of the many mechanisms by which BCL6 abundance and/or activity are deregulated by genetic alterations. This provides strong support for the importance of BCL6 in B-cell lymphoma, but many details remain to be defined. For example, activated B-cell-like (ABC)-like DLBCL tumors transcriptionally align with a stage of development that is similar to plasmablastic B-cells, and express BCL6 protein at a low level. However, genomic studies identified that *BCL6* translocations are more frequent in ABC-like DLBCL tumors compared to the GCB-like DLBCL subtype that more frequently expresses BCL6 at the protein level (Iqbal et al., 2007). Furthermore, high-resolution analysis of DNA copy number in DLBCL tumors revealed that DNA copy number gains of BCL6 were also enriched in the ABC-like DLBCL subtype, but are not associated with increased expression of BCL6 at the transcript level (Green et al., 2014). Two recent comprehensive genetic analyses of primary DLBCL also identified a genetic subset of DLBCL characterized by *BCL6* structural alterations in combination with mutations of NOTCH pathway components and a low rate of SHM-associated mutations suggesting that the tumors had not transited the GC reaction (Chapuy et al., 2018; Schmitz et al., 2018). Mutations in the NOTCH pathway were previously found in low-grade marginal zone lymphomas (MZLs) and transformed MZLs (Flossbach et al., 2011; Spina et al., 2016), leading the authors of the study to speculate that these DLBCL tumors arise from the transformation of an occult MZL. Together, these studies suggest that *BCL6* may function in B-cells outside of its role in orchestrating the germinal center reaction. Characterizing these roles, as well as the complex interplay of epigenetic crosstalk and BCL6 function, will be critical in our understanding of the etiology of B-cell lymphoma and may highlight novel avenues for targeting this critical axis.

## Author Contributions

All authors listed have made a substantial, direct and intellectual contribution to the work, and approved it for publication.

## Conflict of Interest

The authors declare that the research was conducted in the absence of any commercial or financial relationships that could be construed as a potential conflict of interest.
